# Nomogram for predicting postoperative pulmonary complications in spinal tumor patients

**DOI:** 10.1186/s12871-024-02443-7

**Published:** 2024-02-08

**Authors:** Jingcheng Zou, Ge Luo, Liwang Zhou, Xuena Wang, Tingting Wang, Qi Gao, Tao Lv, Guangxin Xu, Yuanyuan Yao, Min Yan

**Affiliations:** 1 Department of Anesthesiology, Second Affiliated Hospital, Zhejiang University School of Medicine, Hangzhou, China; 2 Department of Anesthesiology, The First People’s Hospital of Huzhou, First affiliated Hospital of Huzhou, Huzhou, China; 3 The Fourth Affiliated Hospital, Zhejiang University School of Medicine, Yiwu, China; 4 Key Laboratory of The Diagnosis and Treatment of Severe Trauma and Burn of Zhejiang Province, Hangzhou, China

**Keywords:** Postoperative pulmonary complications, Nomogram, Spinal tumor surgery, Risk factors

## Abstract

**Objectives:**

Although several independent risk factors for postoperative pulmonary complications (PPCs) after spinal tumor surgery have been studied, a simple and valid predictive model for PPC occurrence after spinal tumor surgery has not been developed.

**Patients and methods:**

We collected data from patients who underwent elective spine surgery for a spinal tumor between 2013 and 2020 at a tertiary hospital in China. Data on patient characteristics, comorbidities, preoperative examinations, intraoperative variables, and clinical outcomes were collected. We used univariable and multivariable logistic regression models to assess predictors of PPCs and developed and validated a nomogram for PPCs. We evaluated the performance of the nomogram using the area under the receiver operating characteristic curve (ROC), calibration curves, the Brier Score, and the Hosmer–Lemeshow (H–L) goodness-of-fit test. For clinical use, decision curve analysis (DCA) was conducted to identify the model’s performance as a tool for supporting decision-making.

**Results:**

Among the participants, 61 (12.4%) individuals developed PPCs. Clinically significant variables associated with PPCs after spinal tumor surgery included BMI, tumor location, blood transfusion, and the amount of blood lost. The nomogram incorporating these factors showed a concordance index (C-index) of 0.755 (95% CI: 0.688–0.822). On internal validation, bootstrapping with 1000 resamples yielded a bias-corrected area under the receiver operating characteristic curve of 0.733, indicating the satisfactory performance of the nomogram in predicting PPCs. The calibration curve demonstrated accurate predictions of observed values. The decision curve analysis (DCA) indicated a positive net benefit for the nomogram across most predicted threshold probabilities.

**Conclusions:**

We have developed a new nomogram for predicting PPCs in patients who undergo spinal tumor surgery.

**Supplementary Information:**

The online version contains supplementary material available at 10.1186/s12871-024-02443-7.

## Introduction

Postoperative pulmonary complications (PPCs) have a significant impact on postoperative outcomes, and even mild PPCs are associated with a significant increase in early postoperative mortality, complications, intensive care unit (ICU) admissions, and length of hospital stay [1, 2]. Spinal tumor surgery is characterized by severe surgical trauma, high blood loss, and postoperative complications [3, 4]. It is often accompanied by prolonged surgery, high levels of trauma, nerve damage, airway and respiratory muscle issues, and acute or chronic pain. Previous studies have reported the incidence of PPCs after spinal tumor surgery to range from 5.1 to 16.9% [5–7].

A systematic review identified several prophylactic measures that may reduce the occurrence of PPCs [8], but overuse of these measures can lead to significant financial expenditures and wastage of medical resources. Early identification of patients at high risk of PPCs is an important first step in improving perioperative outcomes and optimizing resource utilization. Therefore, individuals at high risk of PPCs after spinal tumor surgery should receive prompt attention and intervention.

The prediction of PPCs is a primary concern in perioperative medicine. Patient characteristics and associated factors have been found to influence the occurrence of PPCs [2, 9, 10], Although several independent risk factors for PPCs after spinal tumor surgery have been studied [3, 7], a simple and valid predictive model for PPCs following this procedure has not yet been developed. Hence, it is crucial to develop a predictive model for PPCs after spinal tumor surgery based on perioperative variables.

The purpose of this study was to investigate independent predictors of PPCs in patients undergoing spinal tumor surgery and develop a clinical prediction model. Considering the user-friendliness and high accuracy of a nomogram, we converted the resulting model into a nomogram that can be used as a clinical tool for anesthesiologists and surgeons involved in perioperative management. To the best of our knowledge, this is the first nomogram for the assessment of the risk of PPCs in patients undergoing spinal tumor surgery.

## Methods

Ethical approval for this study (reference number 2021 0984) was provided by The ethics committee of the Second Affiliated Hospital of Zhejiang University School of Medicine, Hangzhou, China (Chairperson Prof Zhiying Wu) on 10 November 2021. This retrospective study was reported in accordance with the TRIPOD checklist.

### Participants

Between September 1, 2013, and December 31, 2020, medical record data from patients who had undergone elective spine surgery for spine tumors were retrospectively collected at the Second Affiliated Hospital, Zhejiang University School of Medicine. The inclusion criteria were: (1) being older than 18 years, (2) having a primary diagnosis of a malignant spine tumor, and (3) undergoing general anesthesia with endotracheal intubation and mechanical ventilation. Patients who had undergone ventilation in the preoperative 30 days, had preoperative pneumonia or atelectasis, and had experienced respiratory failure were excluded.

### Data collection

Previous studies [2, 11] noted potential predictors of PPCs include 20 demographic and clinical variables: age, sex, body mass index (BMI), smoking (former or current), alcohol use, American Society of Anesthesiologists (ASA) physical status, preoperative anemia, preoperative hypoproteinemia, origin of tumor, preoperative chemoradiotherapy, spinal tumor location, type of surgery, type of anesthesia, segments involved in spinal surgery, surgical approach, intraoperative blood loss, intraoperative blood transfusion, intraoperative hypotension, intraoperative volume of infused crystalloid, intraoperative volume of infused colloid, and duration of surgery (see details in the Appendix, which is in the Supplement).

### Study outcomes assessments

The primary endpoint of this study was a composite of PPCs that included respiratory failure, prolonged postoperative mechanical ventilation (that occurred more than 48 h after the end of surgery), acute respiratory distress syndrome (ARDS), pneumonia, pleural effusion, and/or atelectasis. Patients with PPCs were identified by reviewing medical records and applying definitions of events agreed upon beforehand (Table S1). Any event identified within the first seven postoperative days was considered a PPC outcome.

### Statistical analysis

We examined patient demographics, comorbidities, preoperative examinations, surgical characteristics, intraoperative characteristics, and PPCs. All variables represent medians (25th, 75th percentiles) or numbers (percentages) unless otherwise specified. Continuous variables, such as age and BMI, were categorized based on assessments following previous studies [9, 12]. Patients with missing values in the variables of interest were excluded from the analysis. The linearity of the remaining continuous variables with the log odds outcome was examined graphically by using the restricted cubic spline (RCS) functions, and non-linear continuous variables were categorized accordingly. Correlations between variables were assessed using the Pearson correlation coefficient, and a heat map was generated to visualize positive and negative correlations. The heat map represents positive correlations in red and negative correlations in blue. The intensity of the color in the heat map is proportional to the strength of the associations between variables.

Univariate logistic regression analysis was used to assess the association between variables and the occurrence of PPCs. Variables with *p* < 0.05 in the univariate analysis, deemed clinically important, and not showing statistically significant associations with other variables, were entered into a multivariate logistic regression model. The final model was developed using both stepwise multivariable logistic regression analysis based on the smallest Akaike information criterion (AIC). Adjusted odds ratios (OR) and their corresponding 95% confidence interval (CI) values were calculated. Collinearity between variables was assessed by using the Variance Inflation Factor (VIF) in the rms package of R, with a VIF ≥ 5 indicating multicollinearity. To avoid overfitting, a rule based on the events-per-variable (EPV) ratio of 10 was applied. The discriminative performance of the prediction model was evaluated using the area under the receiver operating characteristic curve (ROC). An area under the ROC (AUC) of 0.5 indicates no discrimination, while an AUC value ≥ 0.7 suggests moderate discrimination. A calibration curve was used to compare the agreement between the observed and the predicted outcomes. An ideal model provides perfect predictions, resulting in a calibration curve that coincides with the diagonal line. The closer the calibration curve representing the nomogram is to the diagonal line, the better the performance of the nomogram. Additionally, bootstrapping with 1000 resamples was employed to showcase the bias-corrected calibration curve. The performance of the model was further evaluated using the Brier score and the Hosmer–Lemeshow (H–L) goodness-of-fit test. A Brier score greater than 0.3 indicates poor calibration, while a p-value greater than 0.05 from the H–L goodness-of-fit test suggests no significant difference between the predicted and the true values. Internal validation of the prediction model was conducted using bootstrapping with 1000 resamples to calculate an optimism-corrected area under the ROC. Bootstrap resampling is sampling with replacement from the original sample. The performance in the bootstrap sample and original sample represents estimation of the bootstrap performance and test performance respectively. The difference between these performances is an estimate of the optimism in the apparent performance, which is averaged to obtain a stable estimate of the optimism. The internally validated performance is estimated by subtracting optimism from the apparent performance [13].

For clinical use, decision curve analysis (DCA) was conducted to summarize the performance of the model as a tool for supporting decision-making. The net benefit (NB), which is the key component of decision curve analysis (DCA), quantifies the difference between true positives and false negatives [14]. Finally, the final model was converted into a nomogram based on the results of multivariable logistic regression analysis. The regression coefficient (β) of each variable was obtained from the constructed multivariate logistic regression model and proportionally transformed into a score ranging from 0 to 100. The scores corresponding to the variables were summed to obtain a total score, which aligned with the predicted probabilities of PPCs for each patient.

All analyses were performed using R version 4.2.2 (The R Foundation for Statistical Computing, https://www.r-project.org/). The “rms,” “pROC,” “MASS,” “survival,” and “dcurves” R packages were utilized, and 2-sided p-values < 0.05 were considered statistically significant.

## Results

This study utilized data from 526 adult patients who underwent spinal surgery following a primary malignant tumor diagnosis. Among the study subjects, 13 individuals were excluded due to preoperative pneumonia, atelectasis, respiratory failure, and/or received ventilation in the 30 days before the operation (Fig. S1). The distribution of missing variables is visualized in Fig. S2. The proportion of missing values was 4.29%. After excluding patients with missing values in variables of interest, the analysis utilized data from 491 patients. Table 1 presents the demographic and baseline characteristics of the patients, their intraoperative characteristics, and their postoperative pulmonary outcomes. Among the included patients, 61 (12.4%) developed PPCs, 162 (33%) were over 65 years old, 279 (57%) were male, and 157 (32%) had a BMI of 24 kg/m^2^ or higher. Approximately four-fifths (82%) of operations were performed for metastatic lesions.

**Table 1:** Study group characteristics and univariable analysis results for PPCs

Variables	Total patients (*n* = 491)	No PPCs (*n* = 430)	PPCs ( *n* = 61)	Univariate analysis P Value
Age ≥ 65 years, n (%)	162 (33.0)	143 (33.3)	19 (31.1)	0.743
Gender, n (%)				
Male	279 (56.8)	251 (58.4)	28 (45.9)	0.067
BMI ≥ 24 kg/m^2,^ n (%)	157 (32.0)	144 (33.5)	13 (21.3)	0.059
Smoking status, n (%)				
Former	38 (7.7)	33 (7.7)	5 (8.2)	0.980
Current	62 (12.6)	58 (13.5)	4 (6.6)	0.137
Alcohol abuse, n (%)	81 (16.5)	74 (17.2)	7 (11.5)	0.263
ASA ≥ 3, n (%)	97 (19.8)	80 (18.6)	17 (27.9)	0.101
Anaemia, n (%)	203 (41.3)	176 (40.9)	27 (44.3)	0.622
Hypoproteinemia, n (%)	109 (22.2)	93 (21.6)	16 (26.2)	0.426
Origin of tumor, n (%)				
Primary tumor	90 (18.3)	80 (18.6)	10 (16.4)	ref
Secondry tumor from lung	126 (25.7)	117 (27.2)	9 (14.7)	0.314
Secondry tumor from others	275 (56.0)	233 (54.2)	42 (68.9)	0.329
Chemoradiotherapy, n (%)	82 (16.7)	72 (16.7)	10 (16.4)	0.945
Level of tumor, n (%)				
Cervical	85 (17.3)	80 (18.5)	5 (8.2)	ref
Thoracic	178 (36.3)	140 (32.6)	38 (62.3)	0.003
Lumbar	141 (28.7)	128 (29.8)	13 (21.3)	0.373
Sacral	87 (17.7)	82 (19.1)	5 (8.2)	0.970
Type of surgery, n (%)				
Decompression	19 (3.9)	18 (4.2)	1 (1.6)	ref
Tumor resection	89 (18.1)	77 (17.9)	12 (19.7)	0.337
Extensive resection	383 (78.0)	335 (77.9)	48 (78.7)	0.362
Type of anesthesia, n (%)				
Intravenous inhalation combined anesthesia	384 (78.2)	335 (77.9)	49 (80.3)	0.665
Segments, n (%)				
1–2	50 (10.2)	49 (11.4)	1 (1.6)	ref
3–7	411 (83.7)	356 (82.8)	55 (90.2)	0.047
≥7	30 (6.1)	25 (5.8)	5 (8.2)	0.042
Approach, n (%)				
Anterior	58 (11.8)	54 (12.6)	4 (6.6)	ref
Posterior	409 (83.3)	357 (83.0)	52 (85.2)	0.210
Lateral	8 (1.6)	6 (1.4)	2 (3.3)	0.120
Combined	16 (3.3)	13 (3.0)	3 (4.9)	0.168
Blood loss (ml), median [IQR]	800 [400, 1,200]	800 [300, 1,200]	1,000 [800, 1,800]	< 0.001
Blood transfusion, n (%)	304 (61.9)	252 (58.6)	53 (86.9)	< 0.001
Intraoperative hypotension, n (%)	67 (13.6)	57 (13.3)	10 (16.4)	0.514
Crystalloid (ml), median [IQR]	1,500 [1,000, 2,000]	1,500 [1000, 2,000]	2,000 [1,500, 2,500]	0.001
Colloid (ml), median [IQR]	1,000 [500, 1,000]	1,000 [500, 1,000]	1,000 [1,000, 1,500]	0.002
Duration of surgery (ml), median [IQR]	212 [162, 270]	210 [160, 265]	240 [200, 315]	0.002

Variables represent medians (25th, 75th percentiles) or numbers (percentages)

ASA, American Society of Anesthesiologists physical status; BMI, body mass index; IQR, interquartile range

### Data preprocessing

The linearity of each continuous variable with the log odds outcome was assessed using restricted cubic spline functions, and non-linear continuous variables requiring conversion to categorical variables were identified (Fig. S3). The heatmap displayed the correlation analysis results between variables, with color intensity indicating the magnitude of correlation (blue = negative correlation, red = positive correlation). A statistically significant correlation was observed between the intraoperative volume of infused crystalloid and blood loss (Fig. S4, Table S2, and Table S3).

### Predictors of PPCs

Univariate logistic regression was used to identify potential risk factors, and the results are summarized in Table 1. Patients with a preoperative BMI below 24 kg/m^2^ were more likely to develop PPCs, and those who developed PPCs were more likely to receive thoracic tumor surgery, multi-segment spinal tumor surgery, and prolonged surgery. Patients with PPCs exhibited higher levels of intraoperative blood loss, need for intraoperative blood transfusion, and intraoperative volume of infused crystalloid and colloid. Selected relevant factors included BMI, tumor location, segments, blood loss, blood transfused, infused colloid, and duration of surgery. Multivariable logistic regression analysis using both stepwise selection based on AIC revealed four independent risk factors: BMI ≥ 24 [odds ratio (OR), 0.53; 95% confidence interval (CI): 0.27–1.03; *p* = 0.06], tumor location [thoracic tumor (OR, 2.07; 95% CI: 0.71–6.04; *p* = 0.181); lumbar tumor (OR, 0.79; 95% CI: 0.25–2.51; *p* = 0.685); and sacral tumor (OR, 0.34; 95% CI: 0.08–1.46; *p* = 0.145)], blood transfusion (OR, 2.61; 95% CI: 1.11–6.13; *p* = 0.028), and blood loss (OR, 1.0003; 95% CI: 1.0001–1.0006; *p* = 0.016) (Table 2). The variance inflation factors in the model were all less than 5, indicating a low likelihood of collinearity between the variables (Table S4). The linear relationship between continuous independent variables and LogitP is illustrated in Fig. S5, supporting the multivariable logistic regression model.

**Table 2:** Multivariable logistic regression analysis of PPCs

Variables		Unadjusted OR(95% CI)	Adjusted OR(95% CI)		P Value
BMI	< 24 kg/m^2^	Ref	Ref		
	≥ 24 kg/m^2^	0.54 (0.28,1.02)	0.53 (0.27,1.03)		0.06
Level of tumor	Cervical	Ref	Ref		
	Thoracic	4.34 (1.64,11.48)	2.07 (0.71,6.04)		0.181
	Lumbar	1.62 (0.56,4.73)	0.79 (0.25,2.51)		0.685
	Sacral	0.98 (0.27,3.5)	0.34 (0.08,1.46)		0.145
Blood transfusion	No	Ref	Ref		
	Yes	4.08 (1.96,8.5)	2.61 (1.11,6.13)		0.028
Blood loss (ml)		1.0004 (1.0002,1.0007)	1.0003 (1.0001,1.0006)		0.016

BMI, body mass index; CI, confidence interval.

#### Construction and validation of the nomogram for PPCs

**Fig. 1:**
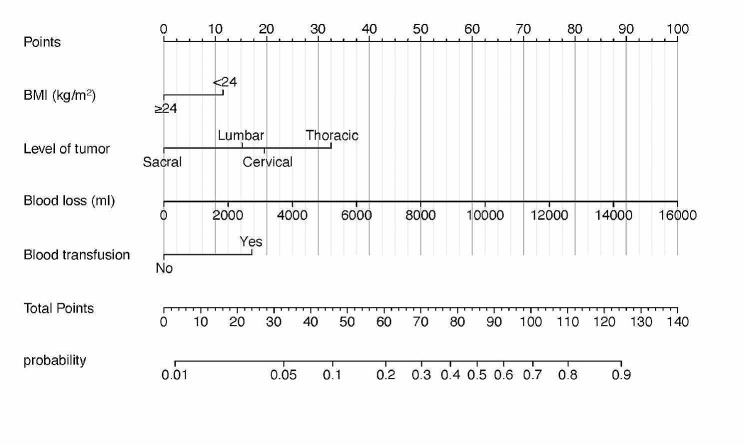
Nomogram for PPCs after spinal tumor surgery. BMI, body mass index

**Fig. 2:**
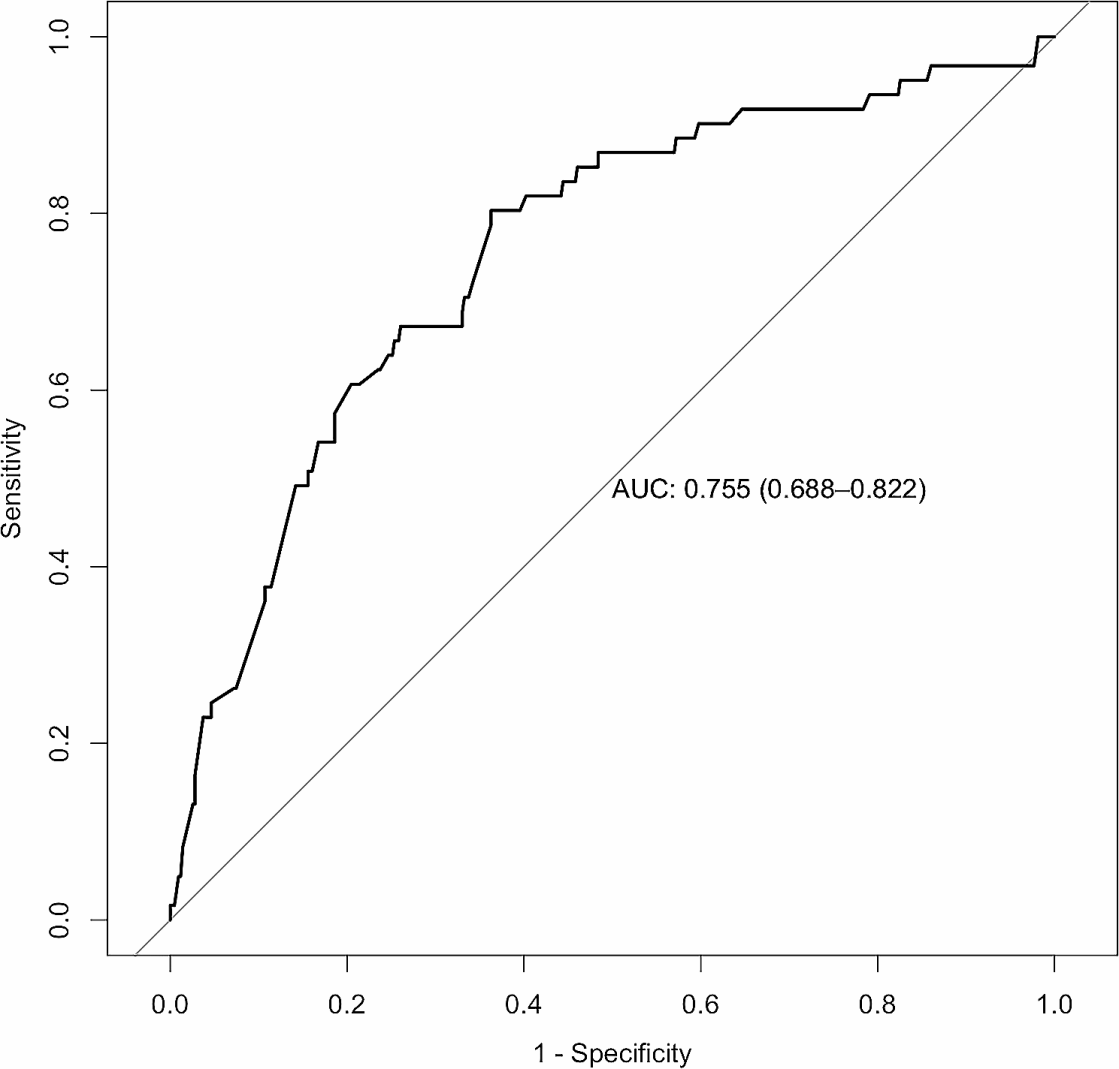
Receiving operating characteristic curves showing the performance of the nomogram in discriminating PPCs

**Fig. 3:**
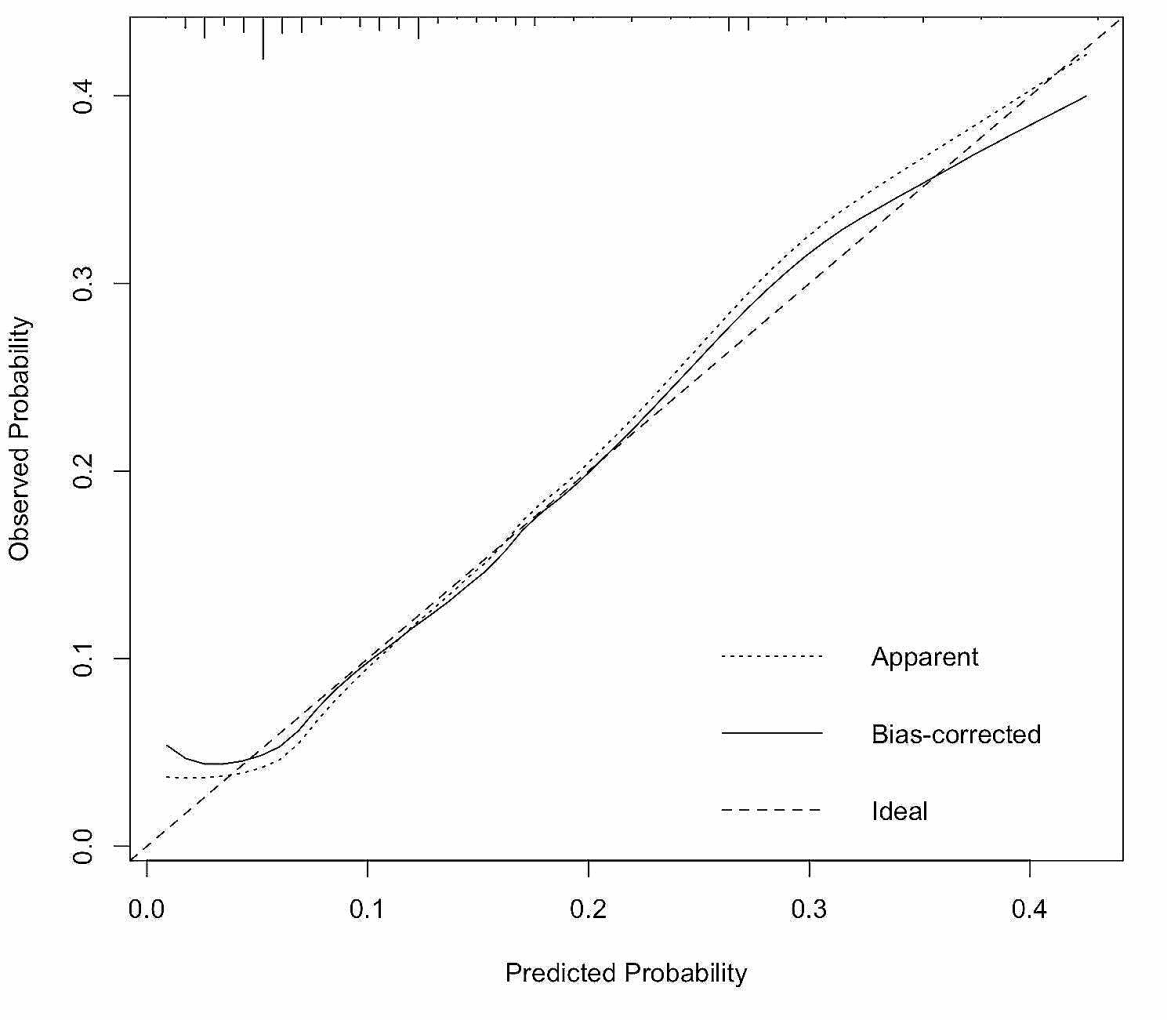
Calibration curves of the nomogram constructed through the bootstrap approach

Four significant risk factors identified through multivariable logistic regression analysis were utilized to create the nomogram model, enabling the calculation of a predictive risk score for each patient (Fig. 1). In the nomogram, intraoperative massive hemorrhage had the highest influence on the occurrence of PPCs, followed by tumor location, blood transfusion, and BMI. The area under the ROC curve (AUC) was 0.755 (95% CI: 0.688–0.822) (Fig. 2). The final model underwent validation, and bootstrap validation resulted in a low optimism value of 0.022. The bias-corrected AUC was calculated as 0.733, indicating the satisfactory performance of the nomogram in identifying PPCs. The calibration curve demonstrated good agreement between predicted and observed values (Fig. 3). Additionally, the Brier score of 0.097 and the H–L Chi-square value of 5.722 (*p* = 0.6783) indicated good calibration of the nomogram.

### Clinical application of the nomogram

**Fig. 4:**
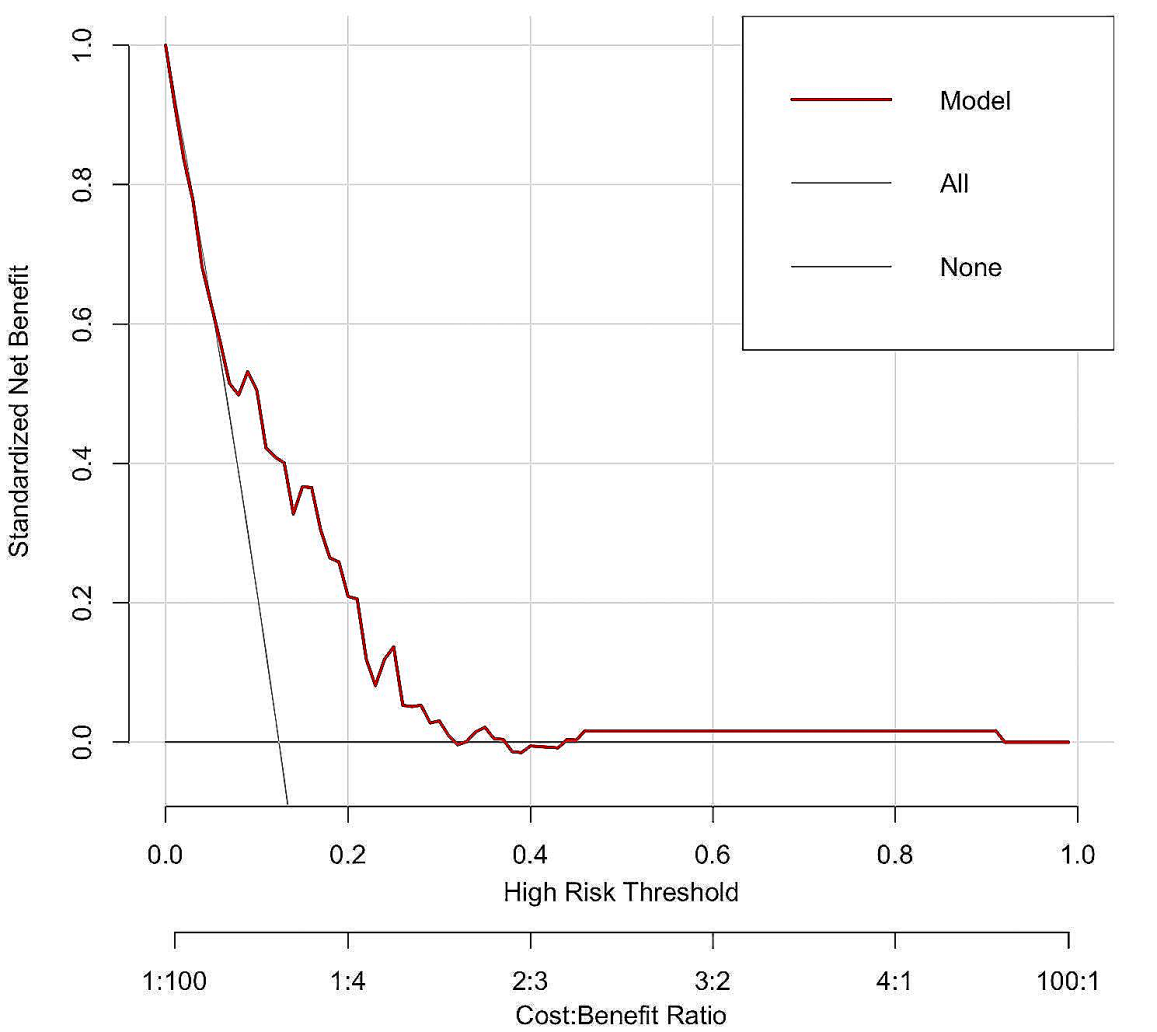
Decision curve analysis of the nomogram

To assess the clinical application value of the nomogram, decision curve analysis (DCA) was conducted to evaluate the net benefit. DCA demonstrated that using the nomogram yielded a positive net benefit at various predicted threshold probabilities, surpassing the default strategies of treating all or no patients (Fig. 4).

## Discussion

Advancements in healthcare, focusing on resource-oriented care and shared decision-making, have raised expectations among clinicians and patients for improved predictions concerning surgical outcomes and associated post-operative complications. Given the significant association between PPCs and poor postoperative outcomes, there is growing demand to reduce the incidence of PPCs following spinal tumor surgery. Additionally, there is an increasing clinical need for a straightforward and effective prediction model specific to a population. The utilization of predictive models can offer care providers valuable insights into high-risk populations and personalized predictions.

In this study, the nomogram included four clinically easily accessible variables: BMI, tumor location, blood transfusion, and amount of blood lost. Our model for PPCs following spinal tumor surgery demonstrated satisfactory discriminative performance in identifying patients at high risk, as evidenced by the area under the ROC curve (AUC) value. Additionally, internal validation indicated good calibration and satisfactory predictive ability of the model for PPCs.

The PPCs risk assessment nomogram revealed that greater blood loss and a higher percentage of transfused blood products were shown to increase the probability of PPCs, aligning with the findings of other studies [2]. Moreover, the study demonstrated that the location of spinal tumors and BMI served as reliable predictors of PPCs in patients undergoing spinal tumor surgery.

In line with the findings of this study, other studies have demonstrated that increased blood loss is associated with major perioperative complications, particularly pulmonary complications [15]. Intraoperative blood loss commonly occurs during surgery for spinal tumors [16]. Consequently, treatments aimed at reducing blood loss would be particularly beneficial to patients undergoing spinal surgery. Given that blood loss is a modifiable factor, several studies have sought to identify interventions that can reduce intraoperative blood loss in spinal tumor surgery. These interventions include the use of tranexamic acid, controlled hypotension [17], pre-operative embolization in patients with spinal metastases from renal cell carcinoma and mixed primary tumor groups [18], and intraoperative cell salvage with leukocyte depletion filters [19]. Different interventions are applied during various perioperative periods as needed. Therefore, clinicians should thoroughly evaluate patients undergoing spinal tumor surgery, predict blood loss, and implement appropriate perioperative measures to reduce PPCs in patients.

Patients who undergo allogeneic red blood cell transfusions during surgery face a higher risk of developing PPCs when compared with those who do not receive transfusions. Previous studies reported similar findings [20, 21], underscoring the significant impact of allogeneic blood transfusions on postoperative complications, including PPCs, in patients undergoing spinal surgery. This heightened risk of PPCs may be associated with transfusion-related acute lung injury (TRALI) [22]. Consequently, clinicians should thoroughly consider potential risks and benefits when making decisions regarding transfusions [23]. For patients undergoing spinal tumor surgery, implementing a more restrictive red blood cell transfusion policy may be necessary. Additionally, appropriate blood management measures such as increasing preoperative hemoglobin levels [24] and utilizing acute normovolemic hemodilution (ANH) [25] should be implemented for patients at high risk of requiring transfusions.

This study incorporated the location of spinal tumors as a variable in the nomogram. Demura et al. reported a statistically higher incidence of PPCs in patients with tumors in the thoracic region compared to those with lumbar tumors [7]. Similarly, Hussain et al. observed patients with tumors in the lumbar and sacral regions had a lower risk of pulmonary complications compared to patients with cervical tumors [6]. These findings are consistent with our study, indicating that spinal tumor location can serve as an important predictor of PPCs. The relatively lower risk of PPCs in the lumbar and sacral spine regions may be attributed to the absence of a close anatomical relationship with the airway or respiratory muscles. Considering the increased risk of PPCs for patients undergoing surgery for thoracic and cervical spinal tumors, enhanced precautions should be taken during these procedures.

Interestingly, this study found that surgical patients with a BMI lower than 24 kg/m^2^ had an increased risk of PPC. While obesity has long been recognized as a risk factor for poor outcomes in various surgical procedures, some studies have described a different effect known as the “obesity paradox” by Mullen et al. [26–29]. Tsang et al. conducted a retrospective study involving 4010 cancer patients with distant metastases and found that, compared to patients of normal weight, being obese or overweight patients was associated with lower all-cause mortality, while being underweight was an unfavorable prognostic factor associated with a higher risk of death [30]. The elevated risk of PPCs following surgery in patients with lower BMI may be attributed to a combination of immunodeficiency and weakness of respiratory muscles in malnourished cancer patients [31]. Williams observed that the increased incidence of adverse events in the low BMI group could be due to the reduced strength of respiratory muscles, inactivity, fatigue, and overall weakness [32]. Molenaar et al. demonstrated the benefits of a rehabilitation program that included a high-intensity exercise regimen three times a week and nutritional intervention, resulting in fewer severe postoperative complications [33]. Therefore, it may be necessary to implement nutritional interventions and improve muscle strength in physically weak patients.

BMI was treated as a dichotomous variable in this study. To investigate the relationship between BMI and the prognosis of tumor patients, Heather et al. conducted an analysis of 22 clinical trials [12]. The study revealed that a BMI of 25 kg/m^2^ was associated with the highest rates of overall survival. The World Health Organization (WHO) has designated a BMI of 25 kg/m^2^ as the cut-off point for individuals of normal weight and overweight. Therefore, this study, which focused on Chinese study subjects, applied a BMI of 24 kg/m^2^ as the cut-off point according to Chinese standard. It is also worth noting that there were no patients with BMI > 35 kg/m^2^ in this study. Therefore, the conclusions drawn from this study are not applicable to patients with moderate and severe obesity. The absence of patients with a BMI > 35 kg/m^2^ in this study may be attributed to cancer-associated weight loss. Changes in BMI among cancer patients could be influenced by the presence of cancer, but due to the retrospective nature of this study, it is challenging to examine this variable comprehensively. Nevertheless, the decrease in BMI reflects the severity of the patient’s condition.

DCA demonstrated that most patients can benefit from employing this nomogram to predict PPCs. Currently, there is no other model available for predicting PPCs following spinal tumor surgery. Decision curve analysis was employed to demonstrate the superiority of the created nomogram over the default strategy. The nomogram can be utilized in a clinical setting to provide risk predictions for individuals and assist clinicians in making intervention recommendations.

This study successfully developed a simple and reliable predictive model for PPCs following spinal tumor surgery. Modifiable and nonmodifiable risk factors were included in this nomogram. For nonmodifiable risk factor (tumor location), we emphasize close observation and early intervention. For modifiable risk factors (BMI, blood loss and transfusion), we suggest establishing a multidisciplinary collaborative strategy to work together to improve the patient’s postoperative pulmonary complications, including two aspects: preoperative prehabilitation and perioperative patient blood management. When evaluating patients in the perioperative period, measures should be taken according to the risk factors in the nomogram. The multidisciplinary strategy is described as follows: Firstly, exercise regimens and nutritional interventions for patients with low BMI, improving hemoglobin levels for patients with anemia, and tumor embolization for patients with indications are need during preoperative preparation phase. Secondly, tranexamic acid, intraoperative cell salvage with leukocyte depletion filters, and acute normovolemic hemodilution should be used at appropriate times. Currently, the conventional diagnosis of PPCs still relies upon clinical observations based on radiology reports. The purpose of developing and using the predictive model is to enhance the identification of high-risk patients immediately after surgery. By utilizing easily assessed perioperative variables, this nomogram could aid doctors in understanding a patient’s risk for PPCs and making appropriate decisions. As a result, high-risk patients may experience improved postoperative outcomes through early interventions such as nebulization, antibiotic therapy, and enhanced recovery pathways. This, in turn, can enhance surgical outcomes and reduce the overall cost of medical care. The prevention of PPCs in cases with major surgery is of increasing interest, and thus, our study of nomogram for PPCs following spinal tumor surgery is of importance.

This study has some limitations. Firstly, being a retrospective study, there may be inherent biases in the collected data. Additionally, the reliability of the nomogram needs to be further confirmed through prospective studies. Furthermore, BMI data were not available for all patients, but the fraction of missing data was low (4.29%) and its absence is unlikely to have had a significant impact on the study results. The data were collected between 2013 and 2020, so the prevalence of PPCs may differ due to advancements in surgical technologies. There is also a possibility of unknown confounding factors. Lastly, this study was conducted in a single center, and it is crucial to conduct external validation using data from other centers.

In conclusion, we have developed a new nomogram for predicting PPCs in patients undergoing spinal tumor surgery. By utilizing this tool, clinicians can implement resource-oriented care and enhance shared decision-making.

### Electronic supplementary material

Below is the link to the electronic supplementary material.

**Supplementary Material 1: Appendix:** Definitions of variables. **Figure S1.** Flow chart of inclusion. **Figure S2.** Distribution of missing values. **Figure S3.** The linearity of continuous variables with the log odds outcome. **Figure S4.** Correlations between variables. **Figure S5.** Analysis of the linear relationship between continuous independent variable and LogitP

**Supplementary Material 2: Table S1.** Definition of postoperative pulmonary complications. **Table S2.** Values of Pearson’s test. **Table S3.** Values of mantel’s test. **Table S4.** Evaluation results of variance inflation factors

## Data Availability

Further inquiries about the datasets used during the current study can be directed to the corresponding author.

## Abbreviations

PPCs: Postoperative pulmonary complications
ROC: Receiver operating characteristic curve
H-L: Hosmer–Lemeshow
DCA: decision curve analysis
C-index: concordance index
ICU: intensive care unit
TRIPOD: Transparent reporting of a multivariable prediction model for individual prognosis or diagnosis
BMI: Body mass index
ASA: American Society of Anesthesiologists
ARDS: Acute respiratory distress syndrome
RCS: Restricted cubic spline
AIC: Akaike information criterion
OR: Odds ratios
VIF: Variance Inflation Factor
EPV: events-per-variable
AUC: Area under the ROC
NB: Net benefit
TRALI: transfusion-related acute lung injury
ANH: Acute normovolemic hemodilution
WHO: World Health Organization
