# Supplementary material for: Nomogram for predicting postoperative pulmonary complications in spinal tumor patients
Source: BMC Anesthesiol. 2024 Feb 8;24:56. doi: 10.1186/s12871-024-02443-7 (PMC10851528; doi:10.1186/s12871-024-02443-7)
Supplement: Supplementary file 2 — Supplementary Material 2: Table S1. Definition of postoperative pulmonary complications. Table S2. Values of Pearson’s test. Table S3. Values of mantel’s test. Table S4. Evaluation results of variance inflation factors [file 12871_2024_2443_MOESM2_ESM.docx]

**Definitions of Variables**

a. Body Mass Index (BMI) was calculated as height(m) * weight(kg)-2.

b. Anemia: for males, hemoglobin < 130g/L; for females, hemoglobin <120g/L.

c. Hypoproteinemia: albumin < 35g/L.

d. Type of anesthesia: intravenous inhalation combined anesthesia or total intravenous anesthesia.

e. Intraoperative hypotension: intraoperative systolic blood pressure is less than or f. equal to 30% of the baseline value, and the duration is greater than 5 minutes or intraoperative systolic blood pressure is less than or equal to 70mmHg, and the duration is greater than 5 minutes.


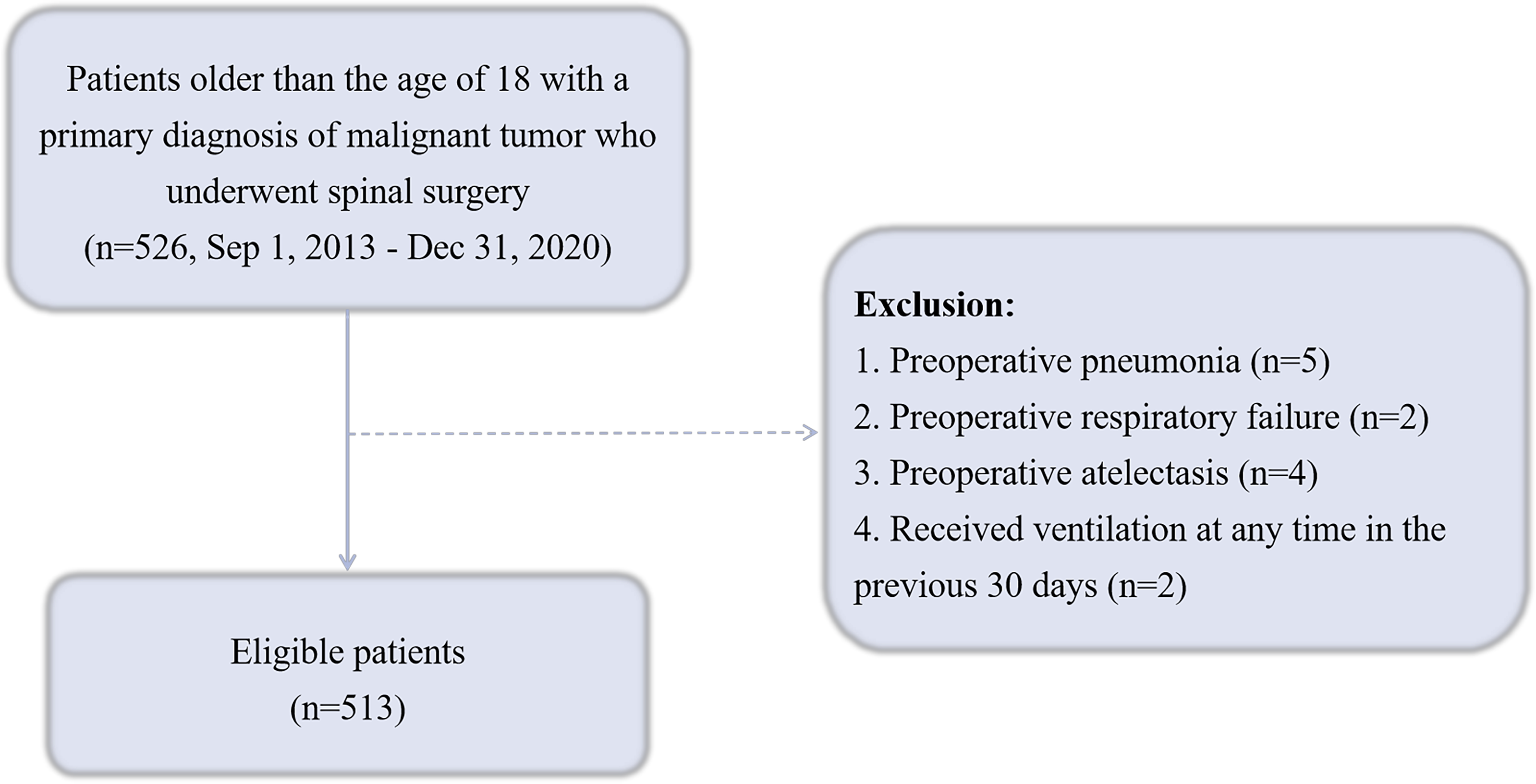


**Figure S1.** Flow chart of inclusion


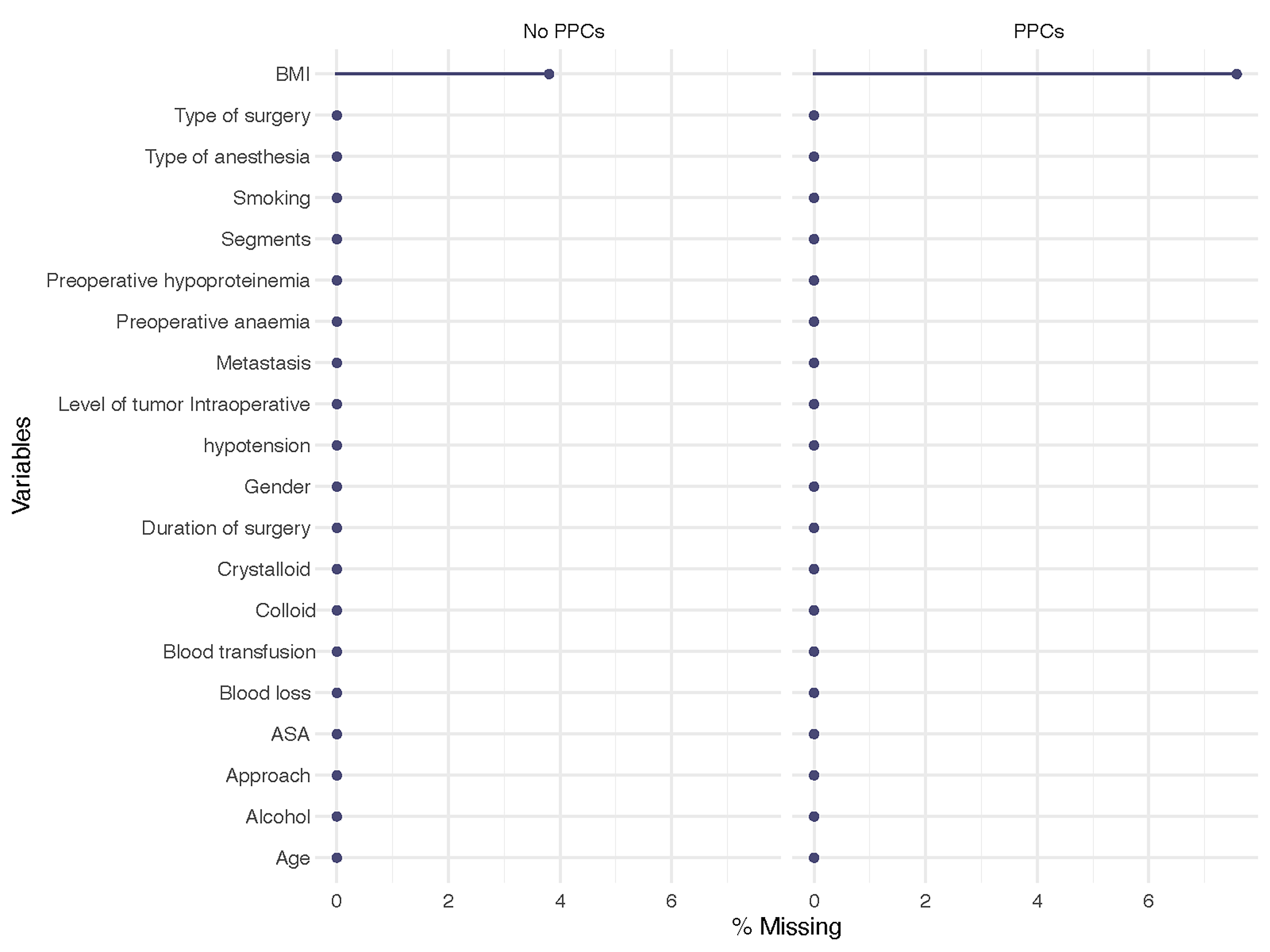


**Figure S2.** Distribution of missing values

BMI, body mass index; ASA, American Society of Anesthesiologists physical status.


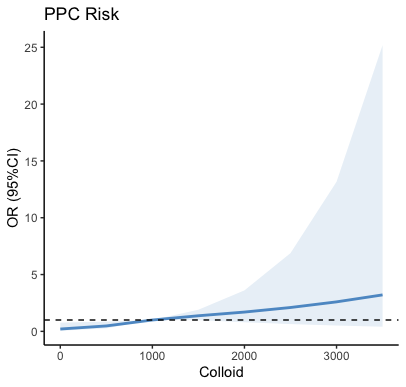

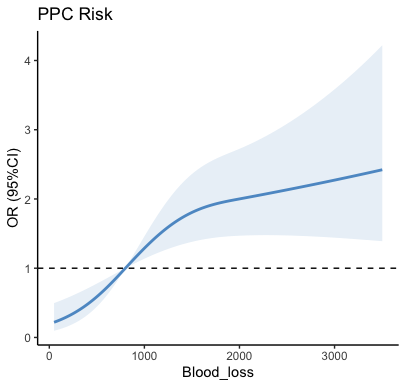

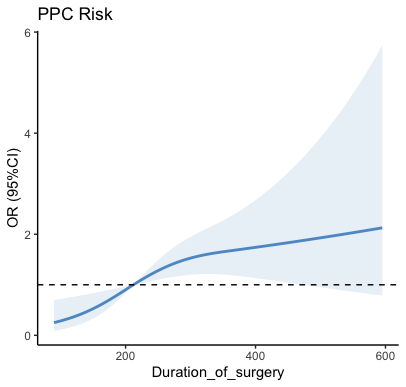

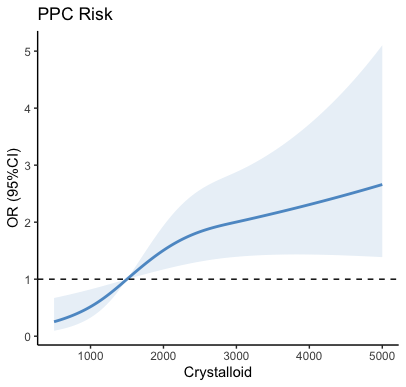


**Figure S3.** The linearity of continuous variables with the log odds outcome


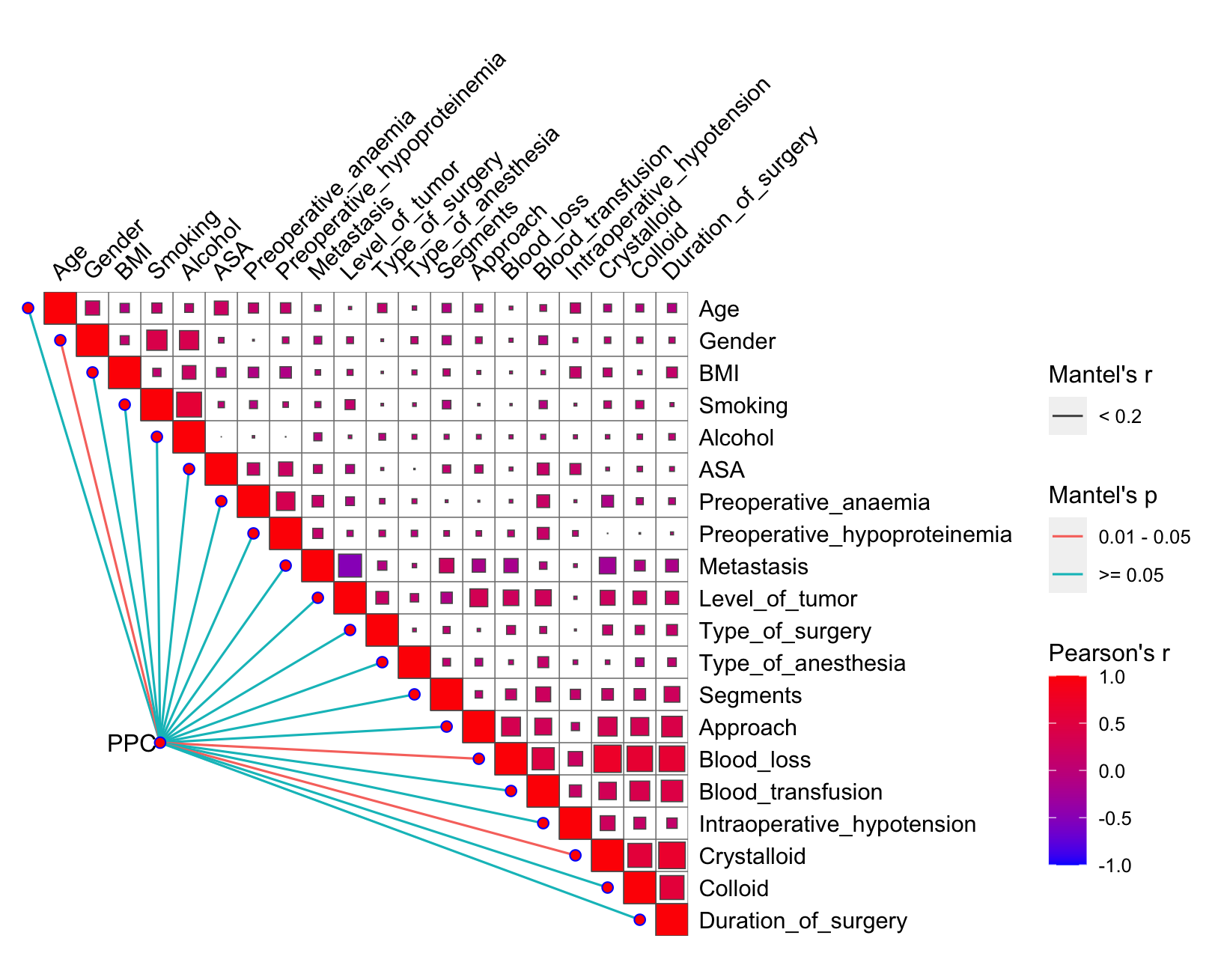


**Figure S4.** Correlations between variables

The correlation is reflected by the color intensity in the scale (blue=negative correlation, red=positive correlation).

BMI, body mass index; ASA, American Society of Anesthesiologists physical status.


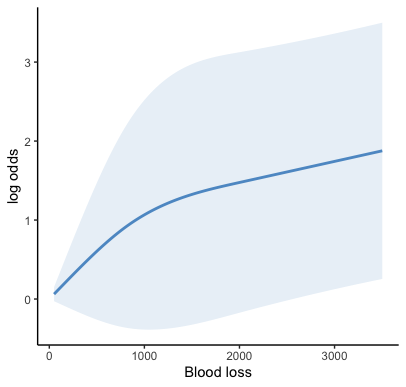


**Figure S5.** Analysis of the linear relationship between continuous independent variable and LogitP (p = 0.319)
